# Family Empowerment (FAME): study protocol for a pilot implementation and evaluation of a preventive multi-family programme for asylum-seeker families

**DOI:** 10.1186/s40814-019-0440-7

**Published:** 2019-04-27

**Authors:** Carlijn M. van Es, Trudy Mooren, Marieke Zwaanswijk, Hans te Brake, Paul A. Boelen

**Affiliations:** 1 Foundation Centrum ‘45, Arq Psychotrauma Expert Group, Nienoord 13, 1112XE Diemen, The Netherlands; 2 Arq Psychotrauma Expert Group, Diemen, The Netherlands; 3 Department of Clinical Psychology, Utrecht University, Utrecht, The Netherlands; 4 Dutch Knowledge Centre for Child and Adolescent Psychiatry, Utrecht, The Netherlands; 5 Impact, Arq Psychotrauma Expert Group, Diemen, The Netherlands

**Keywords:** Asylum-seekers, Families, Multi-family therapy, Family Empowerment

## Abstract

**Background:**

Families applying for asylum have often experienced multiple potentially traumatic events and continue to face stressors during their resettlement. Studies have indicated that traumatic events can negatively impact parenting behaviour and child development. A secondary preventive multi-family intervention programme, called Family Empowerment, was developed. Family Empowerment aims to strengthen parenting skills and prevent exacerbation of emotional problems in asylum-seeker families. This study protocol aims to evaluate the feasibility, acceptability, and potential effectiveness of Family Empowerment to reduce parental mental health problems and improve family functioning.

**Methods:**

An uncontrolled pre-test-post-test design will be conducted, using a mixed-methods approach. Approximately 60 families living at asylum centres and family locations with children aged 0–18 will be included. All participants will be invited to take part in seven sessions of Family Empowerment. Measurements take place at baseline, during implementation of Family Empowerment and 1 week post-Family Empowerment. Demographic data, the quality of the parent-child interaction, family functioning, parental symptoms of depression and anxiety, and participants’ feedback on progress and the therapeutic alliance will be assessed. A programme integrity list will be filled out during each session. Semi-structured interviews at baseline and post-Family Empowerment will be used to evaluate Family Empowerment.

**Discussion:**

This is the first study to provide a pilot implementation and evaluation of Family Empowerment. The current study will inform us on how to improve programme elements and the implementation of Family Empowerment. Limitations are discussed.

**Trial registration:**

Dutch Trial Register, TC = NTR6934. Registered on January 8 2018.

## Background

In 2017, approximately half of all refugees worldwide were minors below 18 years of age. Most of these minors arrived in the country of resettlement with at least one parent or caregiver. These refugee families have been forced to flee their homes as a result of persecution, violence, or conflict [1]. Many of them have been exposed to multiple potentially traumatic events prior to and during their flight, such as the loss of loved ones, war-related events, and physical violence. Furthermore, studies indicate that the continuous stressors refugees are exposed to during resettlement, including social and economic insecurities and long and complex asylum procedures, affect their psychological functioning [2, 3]. Table 1 describes the placement procedure of refugees applying for asylum in the Netherlands.

**Table 1:** Placement of refugee families in the Netherlands

Refugees applying for asylum in the Netherlands first report to an ‘application centre’ where they receive shelter, medical care, and guidance. During the first phase of the asylum procedure, they are accommodated in a ‘process reception centre’. Asylum-seeker families are then placed in an asylum centre that provides them with basic needs, such as food and a roof over their heads, until their asylum application is granted or rejected. Children under the age of 18 have the right to shelter when their asylum application has been rejected. If the application is rejected and the family does not leave the Netherlands within 28 days, families are placed in a ‘family facility’, where they are prepared for deportation. In these centres, families have access to a limited level of facilities [4]. When the children turn 18, their right to shelter ends. As a result of the circumstances in family facilities, including limited financial resources, freedom-restricting measures, and insecurity concerning deportation, an increased level of distress can be expected here [5, 6].	

The accumulation of disruptive events and circumstances before and during the flight and the stressors inextricably linked with migration and resettlement can affect the psychological functioning of asylum-seeker families [2, 7]. Although studies indicate that most refugees do not develop mental health disorders, a substantial minority develops adverse emotional reactions and stress-related complaints, such as difficulty concentrating, sleeping problems, and irritability [8, 9]. Such complaints can impact the daily lives of refugee children, including their educational achievements, social functioning, and family interactions [9, 10].

Asylum-seeker children cannot be seen in isolation from the context and environment in which they grow up. Their parents often have to deal with their own distressing experiences, losses, and continuous stressors. As a result, they are at risk of developing stress-related complaints, which can undermine their parenting skills. For example, they can be less emotionally available, less structuring, and less supporting towards their children [11]. Additionally, exposure to traumatic situations can disrupt the parents’ and child’s capacity to mentalize, which refers to the ability to reflect upon and understand the state of mind of yourself and the other [12, 13]. A reduced capacity to mentalize may negatively affect the development of healthy attachment relationships between parents and their children and consequently affect the overall development of the child [12, 13]. However, clinical experience suggests that the detrimental impact of stress-related complaints on parenting skills can be counteracted by focusing on social and psychological support to help families adapt after distressing events [6, 9]. A recent review showed that studies focusing on preventive programmes addressing asylum-seeker families have been scarce so far [9].

Multi-family therapy (MFT) has been developed by Laqueur et al. [14] in order to improve family functioning and strengthen social support. MFT has since been adapted to meet the needs of various groups, including patients who suffer from symptoms of anorexia, schizophrenia, depression, and conduct disorder [15, 16]. MFT is a psychosocial intervention for a group of at least two families including at least two family members from different generations [17]. Since its development, MFT has been conducted with a variety of theoretical models, illnesses, disorders, and populations [18]. The frequency, duration, and number of sessions differ across settings and populations. Activities are adapted for each specific setting and population. MFT focuses on issues or concerns that families have in common and that are directly or indirectly related to family interactions. Sessions focus on strengthening interfamilial relations. For over 10 years, MFT has been provided to refugee families at the Foundation Centrum ‘45, a Dutch centre for specialist diagnostics and treatment of people with complex psychotrauma complaints.

At the Foundation Centrum ’45, MFT has, so far, mainly been offered to families with severe mental health problems and impaired family functioning. The main principles underlying MFT can also be applied in a preventive programme. Accordingly, therapists and researchers at the Foundation Centrum ‘45 developed a secondary preventive programme for asylum-seeker families based on MFT: Family Empowerment (FAME). FAME addresses families who live under stressful circumstances in asylum centres and family facilities and who may experience the impact of stress-related complaints on parenting, individual mental health, and family functioning. The programme addresses families with diverse cultural backgrounds. As described in more detail below, during FAME, a varying number of five to eight families gather in one room in weekly sessions of approximately 2–3 h for seven consecutive weeks. The aim is to reinforce parenting skills and social support, to improve family functioning and to prevent further development of emotional problems. FAME focuses on activating the families’ own resources and knowledge and allows families to exchange their perspectives, feedback, support, and knowledge. Skills can be developed and practised in a safe environment. Mentalization plays an important role in FAME, as the programme aims to stimulate reflection on one’s own and others’ thought processes and emotions [6].

Although FAME has previously been offered at a family facility, the effects of the programme have not yet been evaluated systematically [6]. Weine [19] proposed a cycle for developing and evaluating preventive programmes for refugee families based on empirical evidence. This cycle encompasses five steps, namely step 0, foundational activities; step 1, template preparation; step 2, situation-specific adaptation; step 3, intervention trial; and step 4, new situations. The development of the FAME manual is in line with both the foundational activities (step 0) and the template preparation (step 1) [6]. The manual describes programme elements that have been developed based on empirical evidence and clinical experience with refugees and MFT [6]. Step 2, situation-specific adaption, was conducted as the programme elements were adapted to fit the needs of asylum-seeker families living in asylum centres and family facilities. The resulting FAME programme adheres to the principles and approaches as described in the manual and incorporates situation-specific adaptations. The aim of the current study is to realize step 3 of the cycle proposed by Weine [19]: performing a pilot intervention trial, to demonstrate programme characteristics such as feasibility (whether the preventive programme is doable), acceptability (whether families and trainers accept the programme), and potential effectiveness (whether the programme coincides with positive changes in key outcomes). This precedes the final step (step 4), namely ‘new situations’, which includes conducting intervention trials at other sites and in other contexts.

### Objectives

The current pilot study aims to evaluate the feasibility, acceptability, and potential effectiveness of FAME to reduce parental mental health problems and improve family functioning. As families living in family locations might suffer from increased levels of distress, we expect these families to have more difficulties concerning the parent-child relationship, parental symptoms, and family functioning than families living in asylum centres. Therefore, baseline distress levels of both categories of families will be compared. Specifically, the objectives are to determine:

1. Whether it is feasible to offer FAME to families living in Dutch asylum centres and family facilities.
2. Whether FAME is acceptable to asylum-seeker families.
3. Whether undergoing FAME coincides with a reduction in parental symptoms of anxiety and depression and improvement in family functioning.
4. Baseline differences and similarities in the parent-child relationship, parental symptoms of anxiety and depression, and family functioning between families living in asylum centres and families living in family facilities.

## Methods/design

### Trial design

An uncontrolled, two-group pre-test-post-test design will be conducted, using a mixed-methods approach. Standardized questionnaires, semi-structured interviews, an observational scale, and a (self-constructed) programme integrity list will be used. The programme, including measurements, will take approximately 10 weeks per group.

### Participants

Study participants will be recruited from the asylum-seeker family population in the Netherlands. Families will be selected through convenience sampling. Eligible families must meet the following criteria: (1) at least one caregiver participates in FAME, (2) at least one child aged 0–18 participates, and (3) the family lives in an asylum centre or family facility. Participants who are not able to function in a group, as reported by health teams of the family facility or asylum centre, are not eligible for this study. For example, participants who are likely to experience difficulties communicating in a group setting as a result of severe psychiatric illness, such as psychosis, will be excluded. Families with psychiatric problems, such as posttraumatic stress disorder or depression, who are likely to be able to benefit from FAME are included in the study. This will be discussed with the health teams prior to inviting the families to take part in the information session about FAME. Each group will include approximately five to eight families. Families will be divided over the groups based on the age of their child (0–5, 6–12, 13–18). However, this division cannot be followed strictly, for example, because some families have children in more than one age category. Therefore, the division will be used merely as a guideline. If there are multiple children in a family, parents are asked with which child they experience most difficulties and will be allocated to the age group of that child. All children of the family are invited to take part in the programme.

As this is a pilot implementation and evaluation of a programme that has not been studied previously, it was not considered appropriate to conduct a reliable sample size calculation. We aim to include approximately five living locations. In each location, we will recruit two groups of approximately six families. This will result in a total sample size of approximately 60 families (5 × 2 × 6). One or two parents and approximately one to two children of each family will take part, resulting in a total number of approximately 90 parents and 90 children.

### Intervention

FAME is offered to families living in family locations and asylum centres. FAME encompasses seven sessions. Apart from the introduction session and final evaluation session, each session has a similar structure. The sessions start with an energizing activity to warm up the participants and promote positive group interactions. Subsequently, the main activity, representing the central theme of the session, takes place. The themes and activities of FAME are based on the metaphor ‘the bucket and the treasure chest’. The bucket is a metaphor for the number of stressful factors and problems families are exposed to. The bucket is filled with soluble and insoluble problems. The treasure chest represents the sources of support the families have. During the programme, families and trainers will focus on questions such as: ‘What are sources of stress in the bucket, and what are sources of support in the treasure chest?’ The bucket and treasure chest can be found in the manual of FAME [6]. The role of the trainer can be described as an ‘eagle’, as he or she walks around the room and zooms in on important positive or problematic interactions that arise. The family members function as ‘consultants’ for each other, as they can offer and receive feedback and support. The sessions are ended by reflecting on what the families have discussed and learned. A manual on FAME for families with children aged six to twelve has been published in Dutch [6]. The programme was further adapted to fit the other age groups addressed in this study. Trainers who offer FAME are therapists working at Centrum ‘45, who have ample experience in working with refugees and asylum-seekers and in working with FAME. Table 2 lists the sessions, themes, and aims of FAME.

**Table 2:** Sessions of FAME

Session	Theme	Aims
1. Introduction	Introduction of FAME, participants, and therapists	Parents know the aim of FAME. Mutual expectations are identified. Methods and framework of FAME are explicated. Parents are introduced to the study. Parents and children feel motivated to take part in the group.
2. Bucket and treasure chest	Stressors and sources of support	Parents are aware of the impact of difficulties on thoughts, behaviour, emotions and relationships. Parents recognize difficulties and risk factors. Parents can distinguish between soluble and insoluble problems. Parents experience mutual recognition. Parents start to develop the following insight: you can do something to decrease stress (locus of control). Families have a positive experience.
3. Impact	Parent-child relationship and the impact of difficulties	Parents are more aware of their own stress reactions. Parents can differentiate between different stress reactions (e.g. rumination, sadness, sleeping problems). Parents are aware of the impact of their stress on the parent-child relationship. Parents realize how they can aid their children. Parents develop an understanding of what they and their children need to facilitate positive development.
4. Tools	Resources and coping; development of the child	Parents gain insight in how to deal with difficulties, and how they are already dealing with difficulties. Parents increase and improve their coping strategies, learn from each other. Parents experience positive interactions with each other. Parents know what helps them to control their own emotions.
5. Discovering	Resources and coping; strengths within the family	Parents become aware of the impact of their own emotions on their child. Parents become aware of how their children perceive the world and emotions of their child.
6. Treasure map	Social support	Parents obtain insight in how they can ask for help. Parents obtain insight in how they can offer help. Parents become more aware of their self-worth.
7. Closing session	Concluding FAME; leave-taking	Looking back: What did you learn? Looking forward: How will you use the things you learned during FAME in the future? Self-confidence of participants is stimulated. Participants develop ideas on how to hold onto and use acquired insights.

Overview of themes and aims for all age groups

### Procedure

Families in both living conditions follow the same procedure. In cooperation with health teams at family facilities and asylum centres throughout the Netherlands, asylum-seeker families will be invited to take part in an introduction session. Participants will be informed about this initial introduction session through flyers and verbal information offered by local partners and researchers. The aim of the introduction session is to explain certain aspects of the programme, such as the structure, number, and duration of the sessions, and to clarify expectations. Families are given the opportunity to ask any questions they might have. During the week after the introduction session, families that have stated their interest in taking part in the programme will be visited. During this visit, any further questions can be answered. Participants who want to take part in the study are then asked to fill in a written informed consent. Parents fill in an informed consent for their children under the age of 16. Minors over 12 years of age fill in an informed consent as well.

Families who agree to take part in the study are subjected to pre-test measures (*t*_1_). One of the researchers will either visit them at home or arrange a quiet room. A professional (telephone) interpreter will be provided. During this visit, parents will be asked to fill out questionnaires (see the ‘Measurements’ section), and a semi-structured interview with the parents will take place. If the participants agree, their semi-structured interviews will be audio-recorded. If the family members agree to take part in videotaping, the parent-child relationship will subsequently be assessed by videotaping the interaction between parent and child for approximately 20 min. Pre-test measures will last approximately 90 min for the parents, and 20 min for the children.

As noted, FAME involves seven weekly 2–3-h sessions. One or two official interpreters will be provided in each group. At the end of each session, the participants are asked to fill in scales measuring their distress and how they rate the session (*t*_2_). The scales will be explained by the trainer, who is aided by the interpreters. Moreover, during the sessions, a researcher will evaluate programme integrity using a predetermined programme integrity list (see below). The final assessment (*t*_3_) takes place in the week after the last session of FAME. The participants will be visited at home or a private room will be arranged. A (telephone) interpreter will be provided. The parents and children aged five and over will take part in individual semi-structured interviews. Parents will fill in questionnaires. Post-test measures will last approximately 65 min for the parents and 30 min for the children. See Table 3 for the schedule of enrolment, intervention, and assessment.

**Table 3:** Schedule of enrolment, intervention, and assessment

		Parents			Children		
Time point	Enrolment	*t*_1_	*t*_2_	*t*_3_	*t*_1_	*t*_2_	*t*_3_
Enrolment							
Eligibility screen	X						
Informed consent	X						
Information session	X						
Assessments							
SCORE-15		X		X			
PHQ-4		X		X			
Semi-structured interview		X		X			X
Demographics		X					
EAS		X			X		
((Y)C)ORS			X			X	
((Y)C)SRS			X			X	
Programme integrity list			X			X	

*t*_1_, pre-FAME; *t*_2_, weekly assessments during FAME; *t*_3_, post-FAME. *EAS* Emotional Availability Scales, *PHQ-4* Patient Health Questionnaire for Depression and Anxiety, *SCORE-15* Systematic Clinical Outcome and Routine Evaluation, *((Y)C)ORS* ((Young) Child) Outcome Rating Scale, *((Y)C)SRS* ((Young) Child) Session Rating Scale

### Measurements

#### Quantitative

##### Parents

Family functioning will be measured using the Systemic Clinical Outcome and Routine Evaluation (SCORE-15; [20]). The SCORE-15 is a 19-item self-report questionnaire that can be used to monitor and report indicators of progress in systemic therapy. It offers an overall measure of family functioning as well as sub-scale scores on the dimensions: strength and adaptability, overwhelmed by difficulties, and disrupted communication. The validity of the SCORE-15 as an index of therapeutic change has been established. The questionnaire demonstrates good test-retest reliability, construct validity, and responsiveness in terms of clinical and reliable change [21, 22].

Parental symptoms of depression and anxiety will be assessed using the 4-item Patient Health Questionnaire for Depression and Anxiety (PHQ-4; [23]). The PHQ-4 has been validated in the general population. The total score is an index of anxiety and depression severity [24].

The following demographics of the participating family members will be collected: age, gender, country of origin, time spent in the Netherlands, number and age of family members, and educational level.

##### Parents and children

Parents and children are subjected to an observational measurement. Quality of the parent-child relationship will be measured using the Emotional Availability Scales (EAS) developed by Biringen et al. [25]. They described emotional availability as ‘the capacity of a dyad to share an emotionally healthy relationship’ (p. 114; [26]). When conducting the EAS, a parent-child dyad is asked to interact as they would usually do for approximately 20 min. These interactions are videotaped and consequently scored on the EAS by certified objective observers with ample experience in working with refugees. EAS measures four caregiver components: sensitivity, structuring, non-intrusiveness, and non-hostility. The child components measured by the EAS are the child’s responsiveness to the caregiver and the child’s involvement with the caregiver. A score on a Likert scale of 1–7 on each component is used for data analysis. A score of 7 suggests that the participant displays optimal behaviours on that scale, a score of 4 indicates inconsistent behaviour, and a score of 1 indicates that the participant displays non-optimal behaviour. Studies suggest that the EAS is universally applicable, and cross-cultural validity has been established in various countries [26, 27].

To monitor participants’ feedback on progress, a self-report scale will be used: the (Young Child) Outcome Rating Scale ((YC)ORS). The (YC)ORS has four single-item subscales: individual, relational, social, and general. Sample questions of the CORS include ‘How am I doing?’ and ‘How are things in my family?’. To assess therapeutic alliance, the (Young Child) Sessions Rating Scale ((YC)SRS) will be used. The four single-item subscales of the (YC)SRS include relationship, goals and topics, approach and method, and overall. Both the (YC)ORS and the (YC)SRS are visual and analogue. Both scales have demonstrated adequate validity, solid reliability, and high feasibility [28, 29].

##### Programme integrity

To evaluate whether the programme is feasible and can be executed as intended, we developed a programme integrity list. This checklist is based on the four dimensions of programme integrity [30]: (1) *Adherence*, the specified components of the programme; (2) *Exposure*, the extent to which family members are exposed to the programme, by monitoring presence and duration (presence is measured by registering the number of minutes each family is present and duration by monitoring the duration of each session in minutes); (3) *Quality of delivery*, therapeutic skills and competence, measured by scoring items such as ‘zoomed in on problematic interactions’ and ‘allowed participants to practise with learned behaviours’; (4) *Participant responsiveness*, measured by assessing reactions during the session, including positive interactions (e.g. laughter) and active participation. Additional questions about participant responsiveness will be asked during the semi-structured interview (*t*_3_). We aim to observe all FAME sessions offered during this study. All assessors will be trained in using the programme integrity list. Two independent assessors will be present during several sessions to fill in the programme integrity list. Inter-rater reliability will be calculated.

#### Qualitative

##### Parents and children

To further investigate the feasibility and acceptability of FAME, semi-structured interviews with parents are held at *t*_1_ and *t*_3_. During *t*_1_, we aim to assess (1) whether participants feel that distressing experiences before, during, and after the flight have impacted their parenting skills and the parent-child relationship, (2) social support, (3) how participants cope with stressors, and (4) expectations concerning FAME. During *t*_3_, we aim to (1) study participants’ evaluations of the programme in terms of usefulness; (2) evaluate programme outcomes: social support, coping strategies, and the parent-child relationship; and (3) evaluate participant responsiveness. Open-ended questions will be posed to the family members. Subsequently, family members score their answers on a 5-point Likert scale. For example, the open-ended question “Which component of the programme was most helpful to you, and why?”, is followed by scoring the question “How helpful was this component?” on a 5-point Likert scale (1 *not helpful* to 5 *very helpful*). The topic list is based on brainstorm sessions with researchers and developers of the FAME programme.

### Statistical analysis

#### Analysis of quantitative data

Quantitative data-analysis will be conducted using SPSS 23 (IBM Statistics). Descriptive statistics of demographic data, the programme integrity list, and the rating scales will be presented for all participants. For continuous variables, means, standard deviations, medians, and ranges will be reported. For categorical variables, numbers and percentages will be reported.

To test the hypothesis that families living at family facilities have more problems concerning the parent-child relationship and family functioning and higher parental symptoms of anxiety and depression than families living at asylum centres, pre-test scores on the EAS, PHQ-4, and SCORE-15 will be compared between these two groups using independent *t* tests.

To evaluate whether undergoing FAME coincides with a reduction in parental symptoms of anxiety and depression and improvement in family functioning, differences between the pre- and post-test scores on the SCORE-15 and PHQ-4 will be calculated. If more than 40 participants have completed the pre- and post-test measures, a mixed-design ANOVA will be executed. However, if there is a large amount of missing data, or if less than 40 participants have completed pre-test and post-test measures, two independent *t* tests will be executed. In addition to statistical significance, it is important to report any meaningful clinical change when studying the impact of an intervention. Calculating the Reliable Change Index (RCI), as proposed by Jacobson and Truax [31], allows us to do so. Using the RCI, we will calculate whether the differences in the scores between *t*_1_ and *t*_3_ are greater than the measurement error. A calculated RCI larger than |1.96| indicates a clinically reliable change with 95% certainty. The RCIs allows us to determine the numbers of participants improved, unchanged, and worsened from *t*_1_ to *t*_3_.

#### Analysis of qualitative data

All audio-recorded interviews will be transcribed verbatim. Data of the semi-structured interviews will be analysed using the qualitative data analysis software programme MAXQDA 10. The current study uses the General Inductive Approach as proposed by Thomas [32]. The approach is often used in qualitative data analysis. Data analysis is guided by the evaluation objectives. Using the General Inductive Approach for analysing qualitative evaluation data, the following five steps will be conducted: (1) initial reading of the text, (2) identifying specific text fragments related to the research questions, (3) labelling fragments to create categories, (4) reducing overlap and redundancy, and (5) describing the most important categories. These steps will result in three to eight outcome categories capturing the key aspects of the most important themes. Reliability of the qualitative data analysis will be assessed by independent parallel coding by two researchers during step 3 and 5.

#### Integrating quantitative and qualitative data

Quantitative and qualitative data from each family will be combined in one document. Findings regarding the evaluation of FAME derived from qualitative and quantitative data will be integrated for each family by analysing whether (1) qualitative and quantitative findings lead to similar or different conclusions and (2) qualitative findings can provide more in-depth information to the quantitative findings. In addition, we will compare the qualitative and quantitative findings of all families that took part in the study and analyse whether any differences or similarities exist.

#### Data management

Each family will be linked to an administration number. The data will be saved under the administration numbers on the protected IT environment of the Foundation Centrum ‘45. The data analysis will be performed at the Foundation Centrum ‘45. The handling of personal data complies with the General Data Protection Regulation.

## Discussion

The current study is designed to evaluate the feasibility, acceptability, and potential effectiveness of FAME, a preventive programme for asylum-seeker families. Moreover, potential baseline differences in the parent-child relationship, parental symptoms of anxiety and depression, and family functioning between families living in asylum centres and families living in family facilities are investigated. Although a large body of research supports the idea that family processes, the parent-child relationship, and the community play a central role in the development and well-being of the child, these processes received little attention in preventive interventions developed for these at-risk families [9]. Moreover, no studies have yet evaluated FAME [6]. This study will inform us on how to improve programme elements and the implementation of FAME.

FAME aims to strengthen and support potential resources within and outside of the families. The current study will indicate whether FAME is a feasible and acceptable programme when offered in a naturalistic setting. An important strength of this study is the inclusion of qualitative data, in addition to various quantitative measures. Conducting semi-structured interviews with the participants enables us to hear the voices of the participants about how they experience the programme and whether they feel it has helped them to deal with the consequences of previous and current stressors.

When executing the proposed study, several barriers can be anticipated. For example, when working with asylum-seeker families, we have to keep into account the many relocations the families are faced with. Families living in asylum centres are often placed in another centre or move away from the centre after the asylum has been granted. Families living at family locations risk deportation. These replacements can result in heightened levels of dropout. Moreover, asylum-seekers are faced with post-migration stressors concerning acculturation and resettlement, such as financial issues, language barriers, and facing an insecure future. These continuous stressors might make it difficult to prioritize preventive programmes such as FAME [3, 9]. To deal with these issues concerning recruitment and inclusion, trainers will collaborate with local health teams in order to reach families, inform them about FAME, and remind them of the sessions as best as possible. To further inform families, the initial information session will focus on disclosing the aim of FAME and the study and clarify mutual expectations. Finally, in order to overcome barriers, FAME is offered at the living locations of the families, diminishing travelling costs and time spent travelling. Nevertheless, the current study will not be able to eliminate dropout as a result of factors such as relocation. These barriers underline the need for studying the feasibility of FAME.

The current study holds several limitations. Because of the small sample size and convenience sampling, the results and their generalizability should be interpreted with caution. Moreover, concerning our objective to establish the potential effectiveness of FAME, we will be unable to attribute any potential effects to FAME as a result of the lack of a control group. However, in line with the developmental cycle proposed by Weine [19], the scope of the current study is not to provide such an extensive evaluation, but to take a next step in the development of a recently developed programme. Possibly, the study will allow us to determine important parameters to estimate the sample size and detectable effect sizes for potential future, larger studies assessing the effectiveness and implementation of FAME.

In conclusion, this is the first study examining the feasibility, acceptability, and preliminary effectiveness of the secondary preventive programme FAME. Our aim is to contribute to the still limited knowledge on preventive programmes for asylum-seeker families. By developing a programme designed to prevent further development of emotional reactions and to improve family functioning, we aim to support these at-risk families preparing for resettlement in a new country, or those facing deportation.

### Trial status

Recruitment commenced in September 2018. The approximate trial duration is 12 months. The trial was registered in the Dutch Trial Register (TC = NTR6934) on January 8 2018 (http://www.trialregister.nl/trial/6723).

## Abbreviations

((Y)C)ORS: ((Young) Child) Outcome Rating Scale
((Y)C)SRS: ((Young) Child) Session Rating Scale
EAS: Emotional Availability Scales
FAME: Family Empowerment
MFT: Multi-family therapy
PHQ-4: Patient Health Questionnaire for Depression and Anxiety
RCI: Reliable Change Index
SCORE-15: Systemic Clinical Outcome and Routine Evaluation
